# The Fusing of Porcelain for Fillings

**Published:** 1906-03

**Authors:** H. M. Semans

**Affiliations:** Columbus, O.


### THE FUSING OF PORCELAIN FOR FILLINGS.

H. M. Semans, D. D. S., Columbus, O.

The exact moment when porcelain is fused is determined only by the individual's idea of what a proper glaze may be, not by some exact calculable flash of time in the procedure of the firing of the porcelain. We may use some one of the many pyrometers placed at our disposal in the dental depots (provided the cash is in sight), even so we depend upon some one's idea of what this finished glaze may be. With some of the later pyrometers it is claimed we are able to be our own judges, and regulate them accordingly. At first thought then a good pyrometer solves the question of how to fuse porcelain, and when it is fused, and that the testing need never be done by eyesight. Well, perhaps so, yet I desire to make this statement: When I am matching a certain shade in a tooth, I am not afraid of baking out colors with my eyes fixed upon the material, I am certain of seeing a glaze that suits my idea of what that glaze should be; to me there is great satisfaction in watching for the glaze, and I use my eyes a great deal more than I do the pyrometer. If one is using a low fusing porcelain it is a very easy matter to see the porcelain melt, and a little experimenting with tablets of porcelain will give the fuse best suited to one's own idea. It is no difficulty at all to see this low melting body change from the dry dead white to the dull dark melted state. Although darkened as in contrast to the muffle glow, yet there is a perceptible glow about it which soon becomes familiar as a timely moment for removal of the fusing mass or else a lowering of the heat. When high fusing material is being used we have greater difficulty to see the melting of the material, owing to the intense glow of the muffle walls and floor, yet a little practice will soon enable one to detect this timely moment also, provided we are equipped with good eyes, and also provided we do not try to stare the interior of the muffle out of countenance. When we look into the clear sky we never stare directly at the sun, our gaze is fixed in its direction with its image impressed upon the eyes without harm, so may we fix our gaze upon the melting object in the bright glow of the muffle without harm to the eyes. In the case of the high fusing bodies, the melting time is seen to approach by a gradual glow, becoming almost if not quite as strong as the surrounding glow, with a beautiful shimmering spreading over its surface. The little roughened outlines are always a guide to this change as they smooth themselves out. Although I may use at times the little melting gold pellet as indicative of the approach of melting time, and although I may know by the seconds how soon after the melt occurs, still I watch for the glow and the shimmer. Never be in a hurry to remove the last bake, also never be in a hurry about introducing the material at any stage of the work into intense heat.

Dry out thoroughly with blotting paper and low heat at all stages before baking, never dry out fast in high heat if you wish to avoid porosity. It is a splendid idea both before and after baking to examine the edges with a magnifying glass to make sure of the exact meet of margins, and the avoidance of slopping over. By far the best polish is that left by the bake; do all your grinding then before the last bake. If foundation body is used the first baking, be careful not to have so much that enamel body can not be used in places that would be on the outside surface of the filling. Always keep in mind that slow cooling means tougher material as contrary to brittleness; that thorough drying out as well as slow drying out, before introduction into the muffle, will avoid porosity.

